# Making persistence assessment work: Now and in the future

**DOI:** 10.1002/ieam.4633

**Published:** 2022-06-29

**Authors:** Johannes Tolls

**Affiliations:** ^1^ Henkel AG & CoKGaA Duesseldorf Germany

The EU Chemicals Strategy for Sustainability aims at improved protection of humans and the environment from harmful chemicals. The protection of drinking water quality is one element of this strategy. To that end, persistent, mobile, and toxic (PMT) or very persistent and very mobile (vPvM) substances are being discussed as a potential new hazard class. This recognition of persistence as an environmentally undesirable property increases the relevance of persistence assessment even further. Recent analyses outlined the improvement needs for experimental persistence and/or degradability testing (Davenport et al., 2022) and advocate for a weight of evidence approach in persistence assessment (Redman et al., 2022) that is more flexible and holistic than the current guidance of the European Chemicals Agency (ECHA) (2017). The present contribution wants to add to the debate by creating awareness of the large number of substances that might be subject to persistence assessments, by reflecting on whether there is sufficient expert capacity available for the overall task and on options for dealing with the issue.

The feasibility question is relevant in view of about 8600 substances registered under Registration, Evaluation, Authorisation and Restriction of Chemicals (REACH) which might qualify as persistent because they fail the criteria for ready or inherent biodegradability or because there are no biodegradation data available. They contrast to approximately 4000 out of the 12 600 substances with a full registration under REACH, which can be considered nonpersistent based on information from biodegradation screening and simulation tests. When taking QSAR (quantitative structure–activity relationship) data as additional information into account the picture changes, but not dramatically. The number of nonpersistent substances increases to ~4900, leaving some 7700 substances that might qualify as persistent. These numbers were established in a recent analysis of the biodegradation data in the ECHA database with interpreted results (accessed via e‐ChemPortal on June 23 and 24, 2021). While this analysis may not be 100% accurate, it nonetheless highlights that for a large number of substances persistence assessments need to be performed. Each of these upcoming assessments might constitute a significant workload for an expert. This is particularly true if these future persistence assessments go beyond equating failing screening tests for ready or inherent biodegradability to persistence. The combination of a large number of assessments and a high workload per single assessment is likely to exceed the available expert capacity. Hence, the overall assessment task not only constitutes a science‐rooted challenge (Redman et al., 2022) but also a practical one.

To address this challenge, I would like to initiate a discussion to identify pathways that enable regulators and the industry to cope with the future persistence assessment for PMT classification. To my mind, this discussion needs to take two perspectives. The first one is establishing a new category of degradability in the environment. I term this category “principally degradable.” It comprises substances that are not readily or inherently degradable and not persistent. As a complement, pragmatic prioritization of substances may be an option to make the workload manageable.

When I consider the criteria for qualifying a substance as “principally degradable,” I have in mind that some 7000–8000 substances are candidates for this category. From my perspective, it is not feasible to subject such a large number of substances to simulation testing. The long test duration and the high cost (due to the need for radiolabeled material) render this impractical. Hence, a science‐based exploration is required to define evidence, which can be considered sufficient to qualify a substance as principally degradable, without reverting to simulation test data. Omitting simulation test data from this approach can be viewed as an element of tiering the persistence assessment. This element may help to focus the use of such costly and long‐lasting tests for substances, which cannot be qualified as principally degradable. Such a qualification requires criteria, which currently are not yet available. I propose case studies for defining such criteria and for starting a discussion on their scientific validity and regulatory usefulness.

The science questions involved in developing relevant criteria include (i) how can multimedia fate modeling be used to direct testing and to put testing results into perspective? (ii) to what extent can read‐across be applied for degradation information? (iii) how can, for example, kinetic information from standard or prolonged screening tests be interpreted with regard to degradability? (iv) which information from nonstandard tests can be considered sufficient to qualify a substance as nonpersistent? and (v) to what extent can evidence of abiotic degradation support in qualifying a substance as nonpersistent? The set of questions may need to be extended and it is clear to me that these questions are also considered when assessing persistence (ECHA, 2017; Redman et al., 2022). In this contribution, however, they are relevant from the perspective of establishing substance as principally degradable.

Exposure‐based prioritization of substances may help in spreading the number of assessments over a longer period of time. It would give high priority to substances that are used in large quantities, at high frequency, and in applications through which significant fractions of the substances are released into the environment. Conversely, substances used in low quantities, at low frequencies, and with low releases to the environment might be addressed at a later point in time because they have a lower risk of building up considerable concentrations in the environment and, finally, in drinking water. In this context, the collection of substance use information under REACH and the availability of specific environmental release categories (Reihlen et al., 2016) as quantitative release estimation information can be very helpful. In addition to exposure, toxicity can be taken into account as well to arrive at a risk‐based prioritization. This requires obtaining drinking water concentrations through measurement or exposure modeling. While this increases the prioritization effort it allows drinking water concentrations to be put into the perspective of the thresholds at which adverse health effects are expected. The smaller the margin between toxicological threshold and measured or modeled exposure concentration, the higher the priority for assessment. Finally, substances which cannot be qualified as principally degradable based on the available evidence may have to be given high priority for further assessment. This may include obtaining additional evidence such as higher‐tier information, preferably originating from simulation tests.

In summary, prioritization of substances appears indispensable to me, be it based on exposure, on risk, or on categorization as principally degradable. Such a prioritization does not interfere with classifying substances as PMT or vPvM based on intrinsic properties. Instead, it will keep the workload of establishing the persistence of a substance feasible for the limited expert resources in agencies and in industry.

In view of the above, it is evident that the science for assessing persistence needs to advance. At the same time, scientists, regulators, and industry need to discuss how to best move ahead with the current state of science. Devising a viable way forward may require careful consideration of regulatory needs, of the full significance of the currently available information on substance emission, exposure, and (bio)degradation profiles, and of the resource constraints in regulatory agencies and industry.

## CONFLICT OF INTEREST

The author declares no conflicts of interest.

## Data Availability

All data can be made available.
